# Loss of Neuropilin2a/b or Sema3fa alters olfactory sensory axon dynamics and protoglomerular targeting

**DOI:** 10.1186/s13064-021-00157-x

**Published:** 2022-01-03

**Authors:** Ryan P. Cheng, Puneet Dang, Alemji A. Taku, Yoon Ji Moon, Vi Pham, Xiaohe Sun, Ethan Zhao, Jonathan A. Raper

**Affiliations:** Department of Neuroscience, University of Pennsylvania School of Medicine, Philadelphia, PA 19104 USA

**Keywords:** Olfaction, Olfactory, Olfactory sensory neuron, Olfactory bulb, Axon guidance, OSN targeting, Odorant map, Protoglomerulus, Zebrafish, Live imaging

## Abstract

**Background:**

Olfactory Sensory Neuron (OSN) axons project from the zebrafish olfactory epithelium to reproducible intermediate target locations in the olfactory bulb called protoglomeruli at early stages in development. Two classes of OSNs expressing either OMP or TRPC2 exclusively target distinct, complementary protoglomeruli. Using RNAseq, we identified axon guidance receptors *nrp2a* and *nrp2b*, and their ligand *sema3fa*, as potential guidance factors that are differentially expressed between these two classes of OSNs.

**Methods:**

To investigate their role in OSN axon guidance, we assessed the protoglomerular targeting fidelity of OSNs labeled by OMP:RFP and TRPC2:Venus transgenes in *nrp2a*, *nrp2b*, or *sema3fa* mutants. We used double mutant and genetic interaction experiments to interrogate the relationship between the three genes. We used live time-lapse imaging to compare the dynamic behaviors of OSN growth cones during protoglomerular targeting in heterozygous and mutant larvae.

**Results:**

The fidelity of protoglomerular targeting of TRPC2-class OSNs is degraded in *nrp2a*, *nrp2b*, or *sema3fa* mutants, as axons misproject into OMP-specific protoglomeruli and other ectopic locations in the bulb. These misprojections are further enhanced in *nrp2a;nrp2b* double mutants suggesting that *nrp2s* work at least partially in parallel in the same guidance process. Results from genetic interaction experiments are consistent with *sema3fa* acting in the same biological pathway as both *nrp2a* and *nrp2b*. Live time-lapse imaging was used to examine the dynamic behavior of TRPC2-class growth cones in *nrp2a* mutants compared to heterozygous siblings. Some TRPC2-class growth cones ectopically enter the dorsal-medial region of the bulb in both groups, but in fully mutant embryos, they are less likely to correct the error through retraction. The same result was observed when TRPC2-class growth cone behavior was compared between *sema3fa* heterozygous and *sema3fa* mutant larvae.

**Conclusions:**

Our results suggest that *nrp2a* and *nrp2b* expressed in TRPC2-class OSNs help prevent their mixing with axon projections in OMP-specific protoglomeruli, and further, that *sema3fa* helps to exclude TRPC2-class axons by repulsion from the dorsal-medial bulb.

**Supplementary Information:**

The online version contains supplementary material available at 10.1186/s13064-021-00157-x.

## Introduction

The precise wiring together of olfactory circuitry is essential for its proper function. Olfactory sensory neurons (OSNs) originate in the Olfactory Epithelium (OE) and send axon projections within the olfactory nerve to the Olfactory Bulb (OB). Each OSN expresses one or a few odorant receptor (OR) genes from a large gene repertoire [1–4]. OSNs predominantly expressing the same OR are stochastically distributed within the OE while their axons converge on a single glomerulus at a stereotypical location within the OB [5, 6]. This pattern of stereotyped convergence converts a diffuse odorant experience into a stereotypical map of neuronal activity in the bulb [7–9]. In the zebrafish, OSNs first project axons to a set of distinct and identifiable intermediate neuropil targets in the olfactory bulb, known as protoglomeruli. Current information suggests that each protoglomerulus is the initial target for a subset of OSNs that all express closely related ORs [10]. The axons in each protoglomerulus then further segregate to form distinct, OR-specific glomeruli [11, 12]. In this report, we examine the contribution that an important family of axon guidance receptors, the neuropilins, play in protoglomerular targeting.

Neuropilins and their ligands, the semaphorins, function in olfactory axon targeting in flies, mice, and zebrafish (Fly: [13–16]) (Mouse: [17–24]) (Fish: [25, 26]) (see discussion for further details). A presumptive whole genome duplication event in teleosts has resulted in duplications of many members of these two gene families [27]. The zebrafish genome contains a pair of orthologs for each of the two mouse neuropilin genes, and one or two orthologs, twelve genes total, for each of the seven mouse class 3 semaphorins [28]. Despite the nearly 400 million years of evolution separating the divergence of fish and mouse ancestral lines, the binding specificities of fish and mouse semaphorin/neuropilin orthologs are remarkably well conserved [28]. Both semaphorin and neuropilin paralogs show distinct expression patterns within the developing zebrafish olfactory system, hinting at a division of labor for each paralog. How the developing nervous system exploits this specialization of paralog expression is an open question.

In tetrapod vertebrates, the main olfactory system and the vomeronasal system are divided, expressing different classes of odorant receptors (ORs as compared to V1R and V2R) and projecting to different areas of the olfactory bulb (the main and accessory olfactory bulbs) [29]. While the tetrapod main olfactory system is predominantly responsible for detecting classical odorants and the vomeronasal system is more specialized for pheromone detection, studies have shown that a subset of both classes of odorants are capable of activating both systems [30, 31]. In zebrafish, there are no anatomically distinct main olfactory and vomeronasal systems, though the molecular components of the two systems are separated and compartmentalized [32]. Main olfactory system type ciliated OSNs and vomeronasal system type microvillous OSNs are mixed together within the OE and can be distinguished by the ORs they express. OSNs expressing traditional ORs co-express Olfactory Marker Protein (OMP), while those expressing vomeronasal type receptors co-express TRPC2 channels. Both cell types extend axons within the olfactory nerve that segregate as they arrive at the OB where they innervate mutually exclusive protoglomeruli [32]. This segregation is maintained in the spatial distribution of OMP-class and TRPC2-class OSN axon glomeruli in the adult olfactory bulb [32]. How these two populations of axons segregate apart from each other and differentially target specific protoglomeruli within the olfactory bulb is unknown.

We conducted an RNAseq-based screen to identify candidate signaling components that might be involved in the differential targeting of OMP as compared to TRPC2 expressing OSNs in the bulb [10]. We compiled a list of candidate genes that are differentially expressed between OMP and TRPC2 OSNs while their axons grow to protoglomerular targets. The classical guidance receptors *nrp2a* and *nrp2b* were identified as candidates more highly expressed in TRPC2-class OSNs in this screen. We investigated the role that nrp2s play in protoglomerular targeting of TRPC2 expressing OSNs. Presumptive loss-of-function mutant lines demonstrate that each of these genes is required for accurate TRPC2 targeting. Through genetic interaction experiments, we show that *nrp2a* and *nrp2b* likely act in parallel to guide TRPC2-class axons to their targets. Further, we identify *sema3fa* as a likely ligand in the same signaling pathway as *nrp2a* and *nrp2b*. Using live imaging, we document TRPC2-class OSN axon guidance in high temporal detail and show that *nrp2a* and *sema3fa* are each required to exclude TRPC2-class OSN axons from the dorsal-medial region of the olfactory bulb.

## Methods

### Transgenic zebrafish lines

Adult zebrafish were raised and maintained according to standard procedures as previously described [33]. All experiments were conducted with the approval of the University of Pennsylvania Institutional Animal Care and Use Committee (IACUC). Veterinary care was supervised by University Laboratory Animal Resources (ULAR). Larvae were staged based on hours post fertilization (hpf) and were raised at 28.5 °C. For some experiments, the 36 hpf time point was obtained by incubating for 1 day at 28.5 °C and 1 day at 25 °C [34]. Tg (omp:lyn-RFP)rw035a and Tg (trpc2:gap-Venus)rw036a transgenic lines were obtained from the Yoshihara laboratory [32]. Tg (omp: GAL4) and Tg (UAS:gap43-citr ine) lines were described by Lakhina et al. [35].

### RNAseq of OMP and TRPC2 neuronal populations

RNAseq was performed as previously described [10]. Single-cell suspensions of 48 hpf OEs were FAC sorted to obtained OMP:RFP-expressing and TRPC2:Venus-expressing cells. RNA was extracted and cDNA was synthesized using a custom oligo-dT primer. One round of in vitro RNA amplification was conducted for OMP-expressing neurons and two rounds for TRPC2-expressing neurons as previously described [36]. Adapter-tagged libraries were synthesized using Illumina TruSeq v2.0 and deep-sequenced on a HiSeq2500 to obtain ~ 100 million reads per sample. Reads were mapped to *Danio rerio* genome assembly GRCz10 using the STAR algorithm [37] and gene counts were generated using Verse [38]. Differential expression analysis was performed using DESeq2 [39].

### In situ probe construction, hybridization, and fluorescent visualization

Single-label in situ hybridization was performed using antisense digoxigenin (DIG) RNA probes as previously described [40]. In situ signals were amplified using a cyanine 3-coupled tyramide kit (TSA Plus Cyanine 3; PerkinElmer, NEL744001KT). Immunohistochemistry, propidium iodide labeling and imaging were performed following tyramide amplification as described below in the immunohistochemistry section.

The plasmids used to make probes for *sema3fa* and *nrp2b* were gifts from the Moens laboratory at the Fred Hutchinson Cancer Research Center, Seattle, WA, USA [41, 42]. For *nrp2a* (refseq accession number NM_212965.1, nucleotides 138–1108) sequences were amplified from cDNA and cloned into pcRII (Invitrogen, K460001) for probe synthesis. Full-length probes were used in all hybridization experiments.

### Zebrafish mutants

The *nrp2a*^p413^ and *sema3fa*^p414^ mutant alleles were generated by introducing a premature stop codon using CRISPR/Cas9 induced mutagenesis to insert a stop codon cassette as previously described [43] using Cas9 protein from PNA-Bio (CP01). Briefly, sgRNA sequences were identified using the CHOPCHOP web tool [44] (*nrp2a* target sequence, 5′-AGAGTGACCTCGGTTTGAGG-3′ ) (*sema3fa* target sequence 5′- GAAGACTCGTGGAACAGAGG-3′). sgRNAs were generated using the pDR274 sgRNA expression vector as previously described [45]. DR274 was provided by Keith Joung (Addgene plasmid # 42250). Corresponding stop codon cassettes were designed and synthesized for both target sites and ordered as ssDNA oligos. Mutagenesis was performed by microinjection of the sgRNA, stop codon cassette, and Cas9 protein into one cell-stage embryos. Stop codon insertion was confirmed by standard PCR methods and sequencing. For *nrp2a*^p413^ an insertion was incorporated into exon 2, after base pair 116. The inserted sequence is (5′ - GTCATGCGTTTAAACCTTAATTAAGCTGTTGTAG - 3′) and introduces a premature stop codon and truncates the protein at position 191 of 927. For *sema3fa*^p414^ an insertion was incorporated into exon 2, after base pair 140. The inserted sequence is (5′ - GTCATGGCGTTTAAACCTTAATTAAGCTGTTGTAG - 3′) and introduces a premature stop codon and truncates the protein at position 43 of 801. Standard PCR-based methods were used to genotype *nrp2a*^p413^ (*nrp2a* forward primer, 5′-CTCCGGGTTTCCCTGACAAG-3′; *nrp2a* reverse primer, 5′-GACCTTCGACCTGGAGAACG-3′) and *sema3fa*^p414^ (*sema3fa* forward primer, 5′-CCCATGCAGGACTGATAAATCTC-3′; *sema3fa* reverse primer, 5′-CCACTGCTTTCCTGTTCAGATT-3′). The *nrp2b*^mn0126GT^ mutant was a gift from the Ekker laboratory at the Mayo Clinic, Rochester, MN, USA and is available from ZIRC [46]. Standard PCR-based methods were used to genotype *nrp2b*^mn0126GT^ (*nrp2b* forward primer, 5′-GCTGAAGATCGGTATCAGACGAAAAACA-3′; *nrp2b* reverse primer, 5′-AGACCTGCCATATTGGTGAGTACCGA-3′; RFP reverse primer, 5′-CCTTGAAGCGCATGAACTCCTTGAT-3′) lines. The *sema3fb*^sa14466^ mutants were acquired from the Sanger Center Zebrafish Mutation Project and obtained through ZIRC. *sema3fb*^sa14466^ is genotyped using a KASP assay (Biosearch Technologies) (KASP sequence: 5′- GAGTTCACAACCWTCACTGTGGATCAGGTCACAGCGGCCGACGGAAACTA [T/G] GAGGTGCTSTTCCTGGGAACAGGTGAGTTTCATGATTTTTTTTTNNNCATGCA − 3′).

### Immunohistochemistry

Immunohistochemistry was performed as previously described [35]. Larvae were fixed in 4% paraformaldehyde in PBS and dehydrated in methanol. Larvae were permeabilized for 30 min in 0.1% collagenase at room temperature. To visualize Citrine-positive axons or Venus-positive axons, larvae were stained with goat anti-GFP (1:300; Rockland Immunochemicals, 600–101-215) and donkey anti-goat IgG Alexa Fluor 488 (1:500; Invitrogen). To visualize RFP-positive axons, larvae were stained with rabbit anti-dsRed (1:300; Clontech, 632,496) and donkey anti-rabbit IgG Alexa Fluor 647 (1:500; Invitrogen). Propidium iodide staining was performed following secondary antibody treatment as described by [47], with the exception that larvae were not treated with RNase. Confocal microscopy was performed on an inverted Leica SP5 using a 63x oil-immersion lens. Z-stacks were acquired through the entire OB with optical sections taken 1 μm apart.

### Quantification of targeting errors

The number of OBs containing OMP- or TRPC2- axons terminating in either individual protoglomeruli or non-protoglomerular regions were counted. Axons were scored as projecting to a particular protoglomerulus only if they terminated in that protoglomerulus and not if they passed though it en route to another location. The percentage of OBs with axon targeting errors was computed and a two-tailed Fisher’s exact test was used to determine statistical significance. The number of ectopic termination sites was computed and a two-tailed Welch’s unequal variance t-test was used to determine statistical significance. Two-tailed Fisher’s exact test wa s used to determine statistical significance of misprojection patterns.

### Live imaging and analysis 

32 hpf larvae were mounted as previously described [48] except in a modified orientation. Briefly, larvae were anesthetized in E3 solution containing PTU and tricaine methanesulfonate until unresponsive and transferred to a 1% low-melting-point agarose (Sigma), E3 solution containing PTU and 80 μg/mL tricaine at 37 °C. Larvae were mounted on a chambered coverslip (μ-Slide 8 Well Glass Bottom - Ibidi #80827) with olfactory pits positioned downward facing against the cover glass, and with bodies at around a 30° angle from the coverslip. Excess agarose was removed and up to 6 total larvae were mounted in the same chamber. The chamber was filled with 1x PTU, 1x tricaine, E3 solution and covered to prevent evaporation. Live imaging was performed on a BioVision spinning-disk confocal microscope system consisting of a Leica DMi8 inverted widefield microscope, a Yokagawa W1 spinning-disk confocal microscope, and a Photometrics Prime 95B scientific complementary metal-oxide-semiconductor camera. Z-stack time series were acquired with VisiView software using a Plan-Apochomat 40x/1.3 Oil-immersion objective and 488 and 561 nm lasers for excitation. Larvae were kept at 28.5 °C for the duration of the experiments in an environmental chamber surrounding the microscope. Z-stacks were imaged at 1 μm sections at 10-min intervals for up to 18 h. Z-stacks were imported into FIJI for reorientation using the “TransformJ Rotate” function to set left and right OE level and olfactory nerve at 15° from vertical. Z-stacks of both hemispheres were cropped and left hemispheres were mirrored for analysis. Live imaging data were analyzed frame by frame to measure cumulative time in the dorsal-medial OB and maximum projection distance into the dorsal-medial OB. Two-tailed Welch’s unequal variance t-test was used to determine statistical significance.

### Segmentation and 3D modeling

In situ segmentation and modeling of *sema3fa* and *sema3fb* expression was performed using Imaris 9.7 (Oxford Instruments). Z-stacks of six hemispheres for each gene were manually aligned using OMP:gal4;UAS:Citirine expression for reference and the OB was manually segmented to create a custom region of interest. In situ signals from all twelve samples were segmented using the “Spot detection” function and aggregated. Segmented objects were filtered for high correlation across samples.

3D modeling of live imaging datasets was performed using Imaris 9.7 for segmentation and FIJI for preprocessing. Time-lapse z-stacks from multiple live imaging experiments were aligned and registered in FIJI using the Fijiyama plugin [49]. Fluorescence intensity of the time-lapse z-stacks were normalized using the “Bleach correction” option in the “Histogram matching” function in FIJI. Axon positions were extracted by manual thresholding and converting to binary. Registered binary time-lapse z-stacks were averaged to create time-lapse probability maps of axon positions. Time-lapse pro bability maps were averaged along the time-axis to create probability maps of axon positions for the live imaging experiment. The “Surface” function in Imaris was used to segment the probability maps at defined intensities.

## Results 

### Expression patterns of *nrp2a*, *nrp2b*, and *sema3fa* in the developing zebrafish olfactory system

**Fig. 1:**
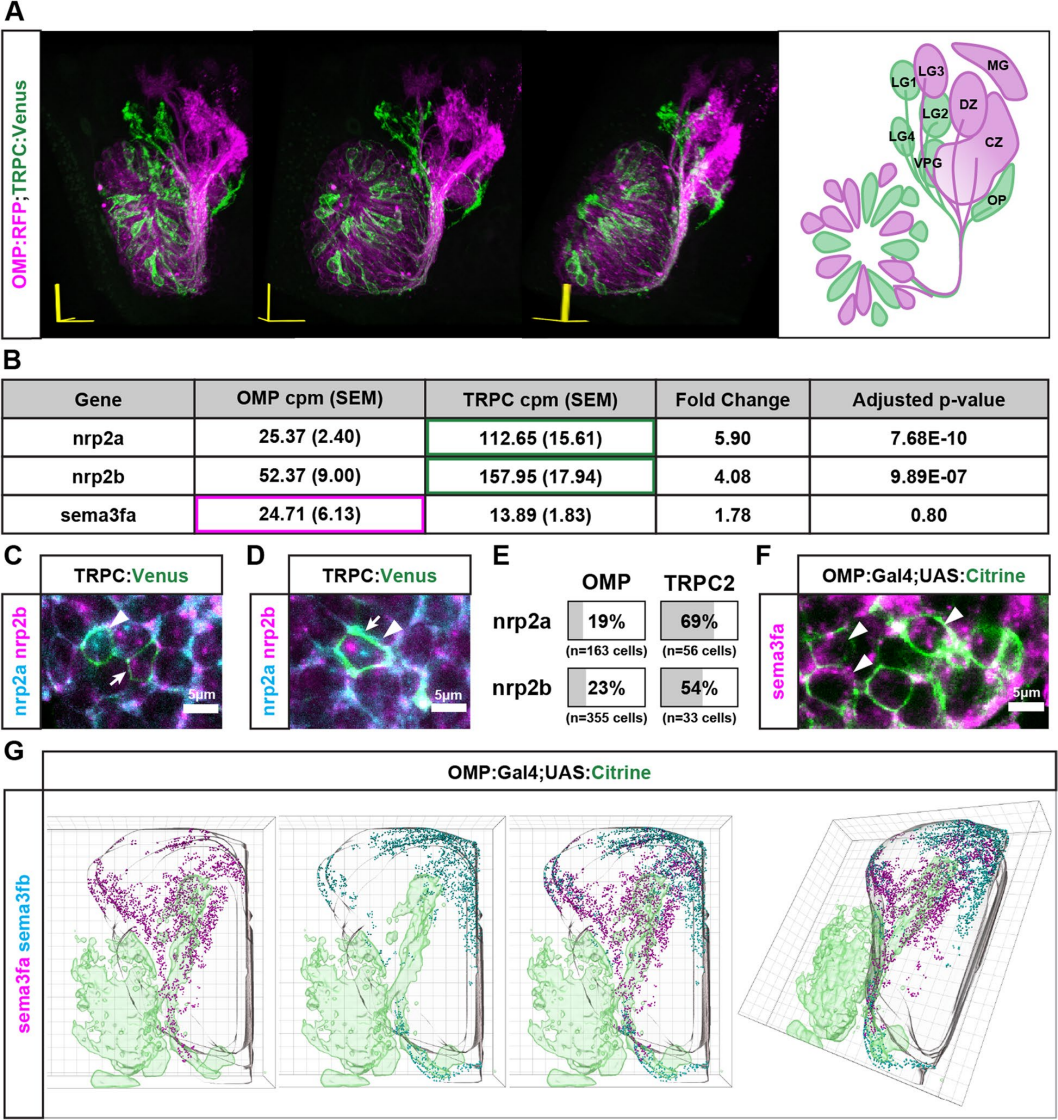
Expression patterns of *nrp2a*, *nrp2b*, and *sema3fa* in the developing zebrafish olfactory system. **A.** 3D projection and schematic representation (frontal view) of an optical z-stack of the 72 hpf olfactory system. One side of the olfactory system is shown with dorsal up and lateral on the left. OSNs are labeled by OMP:RFP (Magenta) and TRPC2:Venus (Green). OMP-expressing axons project to the central zone (CZ), dorsal zone (DZ), lateral glomerulus 3 (LG3), and medial glomerulus (MG). TRPC2-expressing axons project to the olfactory plexus (OP), lateral glomerulus 1,2, and 4 (LG1, LG2, LG4), and the ventral posterior glomerulus (VPG). **B.** RNAseq expression data from four replicate experiments for *sema3fa*, *nrp2a*, and *nrp2b* in OMP and TRPC2 expressing OSNs. **C.** *Nrp2a* mRNA (cyan, arrowhead) and *nrp2b *mRNA (magenta, arrow) is detected independently in the cell bodies of TRPC2:Venus labeled cells (green) in the OE at 48 hpf. **D.** *Nrp2a* mRNA (cyan, arrowhead) and *nrp2b* mRNA (magenta, arrow) is detected in the same cell body of TRPC2:Venus labeled expressing cells (green) in the OE at 48 hpf. **E.** Percentage of OMP and TRPC2 expressing cells with *nrp2a* or *nrp2b* expression. **F.** *Sema3fa* mRNA (magenta) is detected in the cell bodies of OMP:Gal4;UAS:Citrine expressing cells (green) in the OE at 48 hpf. **G.** *Sema3fa* and *sema3fb* expression in the olfactory system of 36 hpf zebrafish. 3D model of *sema3fa* mRNA (magenta) and *sema3fb* mRNA (cyan) distribution within the OB (grey outline). *Sema3fa* is concentrated in the anterior and dorsal-medial regions of the OB and *sema3fb* is concentrated in the dorsal-medial regions of the olfactory bulb. Data aggregated from 6 OB hemispheres for *sema3fa* and 6 OB hemispheres for *sema3fb*

A striking feature of the zebrafish olfactory projection is that axons originating from OMP-expressing OSNs and from TRPC2-expressing OSNs terminate in distinct and separate protoglomerular neuropils in the OB (Fig. 1A, Additional file 1). The guidance mechanisms controlling this differential targeting are unknown. We reasoned that axonal guidance receptors that are differentially expressed in OMP-class as compared to TRPC2-class OSNs are candidates for mediating this differential targeting. We used an RNAseq-based approach to identify candidate axonal guidance receptors expressed at different levels in these two classes of OSNs. Main olfactory OMP-class OSNs and ‘vomeronasal’ TRPC2-class OSNs were separated and collected by FAC sorting from dissociated cell suspensions prepared from the heads of transgenic OMP:RFP; TRPC2:Venus 48 hpf embryos. At this developmental stage, axons of both classes of OSNs are actively extending into the olfactory bulb. Bulk RNAseq was performed separately on OMP-class and TRPC2-class OSNs in quadruplicate [10], and differentially expressed genes were identified by DESeq2 [39]. The canonical guidance receptors *nrp2a* and *nrp2b* were found to be expressed at a higher level in TRPC2-class as compared to OMP-class OSNs (Fig. 1B). In situ probes for *nrp2a* and *nrp2b* mRNAs confirm their enhanced expression in TRPC2-class OSNs at 48hpf (Fig. 1C-E). We found that *nrp2a* and *nrp2b* RNAs are detected in overlapping subsets of TRPC2-class OSNs (Fig. 1D).

To identify potential neuropilin ligands that might serve as TRPC2-class axonal guidance cues, we first examined class 3 semaphorins that preferentially bind *nrp2s*. A variety of class 3 semaphorins can bind and signal through holoreceptors composed of Nrp2 and PlexinAs [50]. Previous binding studies in vitro showed that Sema3fa, Sema3fb, Sema3ga, and Sem a3gb all preferentially bind to either Nrp2a or Nrp2b as compared to Nrp1a or Nrp1b [28]. We probed the developing olfactory system for a variety of class3 semaphorins by in situ hybridization of whole mount heads (data not shown). One of these candidate ligands, *sema3fa*, was expressed in OMP-class OSNs (Fig. 1F). *Sema3fa* mRNA was detected on average at a higher level in OMP-class as compared to TRPC2-class OSNs by RNAseq, although there was too much variance between replicates for the difference to be considered significant (Fig. 1B). *Sema3fa* mRNA is also detected in the anterior and dorsal-medial regions of the 48 hpf OB (Fig. 1G).

### *Nrp2a*, *nrp2b* are required for normal protoglomerular targeting of TRPC2-class OSNs

To test whether *nrp2a* or *nrp2b* are required for normal protoglomerular targeting, we generated a mutant allele for *nrp2a* by CRISPR/Cas9 knock in of a multi-reading frame stop codon cassette [43] and took advantage of an existing gene-trap mutant allele for *nrp2b* [46]. Each of these alleles are likely nulls since they introduce premature stop codons into early exons that cannot be skipped without shifting the frame of translation. These mutants were crossed into fish incorporating OMP:RFP and TRPC2:Venus transgenic reporters to generate *nrp2a*+/−;OMP:RFP;TRPC2:Venus or *nrp2b*+/−;OMP:RFP;TRPC2:Venus lines. These lines were then crossed to themselves to determine how the loss of either *nrp2a* or *nrp2b* would affect protoglomerular targeting. Embryos were collected at 3 days post fertilization (dpf), genotyped, and processed for imaging. Confocal optical slices through the entire olfactory bulb were used to reconstruct OMP-class and TRPC2-class OSN axon trajectories. Counterstaining with propidium iodide labeled nuclear material in all cell bodies, and its absence allows the visualization of individual protoglom erular neuropils. The fidelity of protoglomerular targeting was assessed by scoring for the presence of OMP-class or TRPC2-class OSN axon termini in each individual protoglomerulus. Termini present in non-protoglomerular regions of the OB were also noted. Scoring was performed independently by two individuals blind to the genotypes of the embryos.

**Fig. 2:**
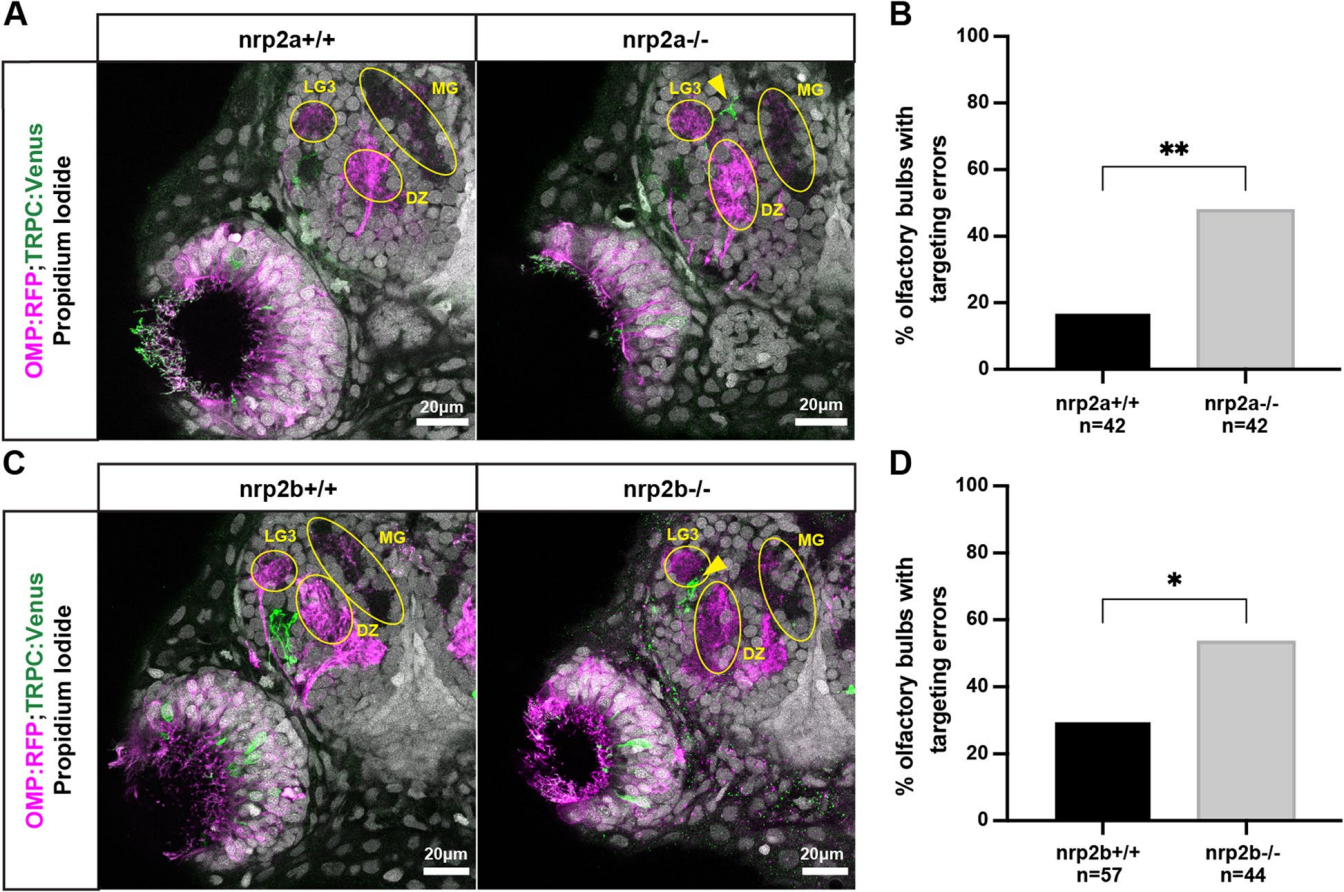
*Nrp2a* and *nrp2b* are required for normal protoglomerular targeting of TRPC2-class OSNs. **A.** Representative confocal sections of wild type and *nrp2a* mutant siblings. Yellow arrows indicate misprojecting TRPC2-class axons. **B.** The percentage of olfactory bulbs with targeting errors is higher in *nrp2a* mutants as compared to wild type siblings. **C.** Representative confocal sections of wild type and *nrp2b* mutant siblings. Yellow arrows indicate misprojecting axons. **D** The percentage of olfactory bulbs with targeting errors is higher in *nrp2b* mutants as compared to wild type siblings

*Nrp2a* mutant animals had a higher rate of misprojecting TRPC2-class OSN axons as compared to wild type siblings (Fig. 2A,B). TRPC2-class axons misprojected to an average of 0.476 ectopic locations per OB hemisphere, a statistically significant increase relative to the 0.167 error rate in wild type siblings. These misprojections tended to concentrate in the MG protoglomerulus, anteriorly, and other non-protoglomerular regions (Fig. S1A). Nrp2b mutant animals also had a statistically higher rate of misprojecting TRPC2 expressing OSNs as compared to wild type siblings (Fig. 2C,D). TRPC2-class axons projected to an average of 0.533 ectopic locations per OB hemisphere as compared to a 0.294 error rate in their wild type siblings. These misprojections were concentrated in the LG3 protoglomerulus (Fig. S1B).

### *Nrp2a and nrp2b* act in a partially redundant, parallel signaling pathway

**Fig. 3:**
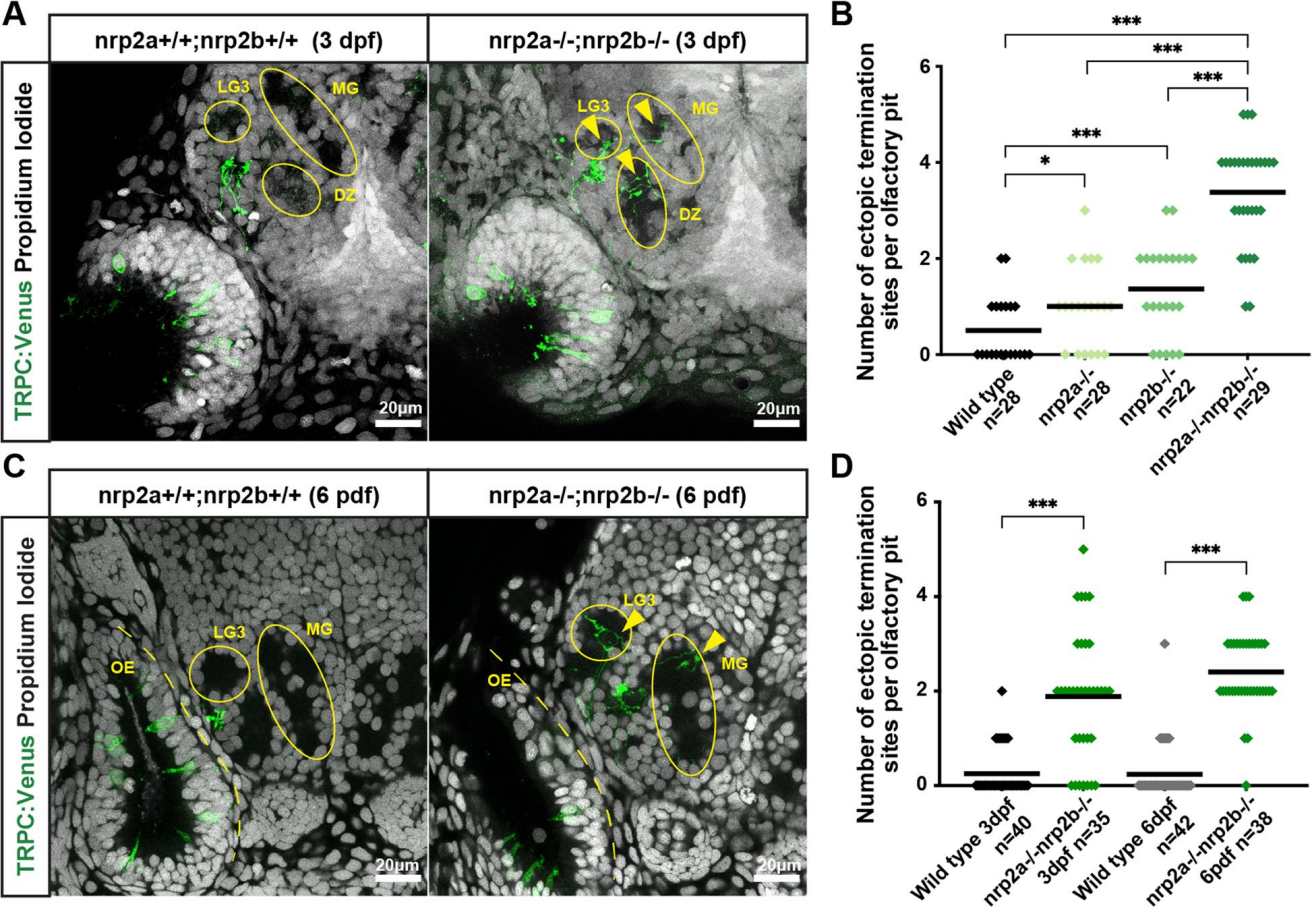
*Nrp2a* and *nrp2b* likely work in parallel and targeting errors persist for days. **A.** Representative confocal sections of 3 dpf wild type and *nrp2a;nrp2b* double mutant siblings showing multiple misprojections by TRPC2-class OSNs in the MG, DZ, and LG3 protoglomeruli. Yellow arrows indicate misprojecting axons. **B.** TRPC2-class OSNs in *nrp2a;nrp2b* double mutants project axons to more ectopic termination sites than single mutant or wild type siblings. **C.** Representative confocal sections of 6 dpf wild type and *nrp2a;nrp2b* double mutant siblings showing multiple misprojections by TRPC2-class OSNs in the MG and LG3 protoglomeruli. Yellow arrows indicate misprojecting axons. **D** Targeting errors of TRPC2-class OSNs persist into later stages of development. The number of ectopic termination sites per olfactory pit is significantly higher in *nrp2a;nrp2b* double mutant as compared to wild type siblings at both 3 dpf and at 6 dpf

*Nrp2a* and *nrp2b* are paralogs that are orthologous to mammalian * Nrp2*. The two genes have overlapping expression within the TRPC2-class of OSNs. Since the phenotype for neither mutant allele was fully penetrant, it is possible that *nrp2a* and *nrp2b* serve partially redundant functions in regulating protoglomerular targeting of TRPC2-class OSNs. To further test this idea, we generated *nrp2a*+/−;*nrp2b*+/− fish containing the OMP:RFP and TRPC2:Venus transgenic reporters. These fish were crossed together to generate siblings of all genotypes ranging from wild type to *nrp2a*;*nrp2b* double mutants. The fidelity of protoglomerular targeting was not significantly different in *nrp2a*+/−;*nrp2b*+/− trans-heterozygotes as compared to wild type siblings (not shown). The *nrp2a*−/− and the *nrp2b*−/− single mutant animals replicated the misprojection phenotypes observed in the previous single mutant experiments (Fig. 3A,B). *Nrp2a*−/−;*nrp2b*−/− double mutant animals had a statistically significant enhancement of the TRPC2-class OSN misprojection phenotype as compared to either single mutant alone, and this phenotype was nearly fully penetrant (Fig. 3A,B).

Quantifying the percentage of OB hemispheres with misprojections is a sensitive measure of infrequent misprojections at a population level; however, this measurement becomes saturated when used on highly penetrant phenotypes. To better represent the more robust misprojection phenotype in the* nrp2a*−/−;*nrp2b*−/− double mutants, we counted the number of inappropriate protoglomerular and non-protoglomerular targets (ectopic termination sites) occupied by TRPC2-class OSNs in each sample. TRPC2-class OSNs in double mutants projected to a significantly higher number of ectopic termination sites as compared to their single mutant or wild type siblings. Since targeting errors in single mutant or wild type embryos are nearly always comprised of single axons, while multiple overlapping axons sometimes contribute to the errors in double mutant embryos, our scoring underrepresents the number of misprojecting axons in the double mutant samples. Despite this relative undercounting, TRPC2-class axon misprojections were greater in double mutants (3.38 inappropriate protoglomerular targets per OB hemisphere) than in *nrp2a* and *nrp2b* single mutants combined (1 and 1.3 = 2.3 inappropriate protoglomerular targets per OB hemisphere). Significantly higher rates of misprojections to the OMP-class CZ, DZ, LG3, and MG target protoglomeruli were observed in double mutants as compared to the two single mutants combined (Fig. S1D). This error enhancement in the double mutants may be more than purely additive, suggesting that *nrp2a* and *nrp2b* act in a partially redundant, parallel signaling pathway to regulate protoglomerular targeting of TRPC2-class OSN axons.

Next, we asked whether the misprojection phenotype in *nrp2* mutant embryos represents a lasting alteration to olfactory circuitry or if targeting errors are corrected as development progresses. Wild type and *nrp2a*;*nrp2b* double mutant sibling 3 and 6 dpf embryos were compared. There was a small but statistically significant increase in the extent of misprojections observed at 6 dpf as compared to 3 dpf in double mutants (Fig. 3C,D). This small increase in error rate might be explained by increasing numbers of TRPC2-class OSN axons innervating the bulb at the later developmental age. Nevertheless, these observations suggest that TRPC2-class OSN misprojections are not corrected during larval development.

### *Sema3fa* is required for normal TRPC2-class OSN protoglomerular targeting

*Sema3fa* is a member of the Class 3 semaphorin family and has been shown to bind with Nrp2a and with Nrp2b in preference to Nrp1a or Nrp1b [28]. It is expressed in OMP-class OSNs (Fig. 1F) and in situ probes for *sema3fa* mRNA detected its expression in the anterior OB in a region that extends into the dorsal-medial OB at 36hpf (Fig. 1G). To test whether *sema3fa* is required for normal protoglomerular targeting, we used CRISPR/Cas9 to generate a *sema3fa* mutant allele following the protocol described in Gagnon et al. 2014 [43]. The resulting allele is likely a null because the inserted stop codon cassette introduces an in-frame premature stop codon in an early exon that cannot be skipped without putting the translation product out of frame. It was crossed into fish containing OMP:RFP; TRPC2:Venus and the progeny of crosses between these fish were assessed for protoglomerular targeting fidelity as previously described. TRPC2-class OSNs in *sema3fa*−/− animals had an increased rate of misprojections compared to wild type siblings. TRPC2-class OSNs misprojected to 0.67 incorrect targets per OB hemisphere in *sema3fa* mutants as compared to 0.13 incorrect targets in wild type siblings. The misprojections mostly consisted of targeting errors into CZ and LG3 protoglomuli. 

**Fig. 4:**
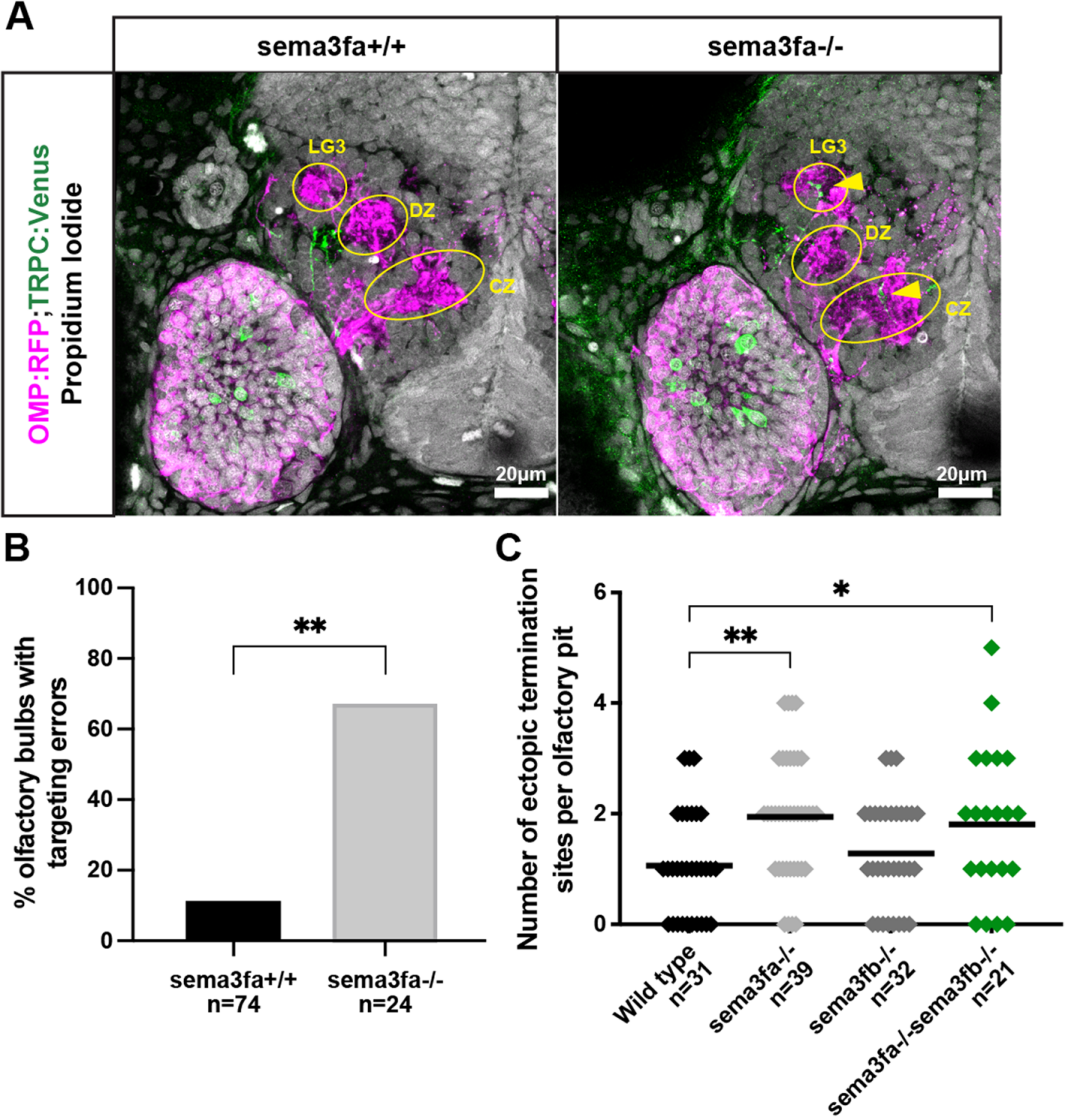
*Sema3fa* is required for normal TRPC2-class OSN protoglomerular targeting. **A.** Representative confocal sections of wild type and *sema3fa* mutant siblings. Yellow arrows indicate misprojecting axons. **B.** The percentage of olfactory bulbs with targeting errors is higher in *sema3fa* mutants as compared to wild type siblings. **C.** The misprojection phenotype of *Sema3fa* mutants is not significantly different from *sema3fa*;*sema3fb* double mutants

While *sema3fa* mutants show increased rates of misprojections of TRPC2-class OSNs (1 inappropriate protoglomerular targets per OB hemisphere), the misprojection phenotype does not fully account for the much more severe *nrp2a*−/−;*nrp2b*−/− phenotype (3.38 inappropriate protoglomerular targets per OB hemisphere). This suggests that other Nrp2 ligands are involved in *nrp2*-dependent OSN axon guidance. One possible candidate is the Nrp2a- and Nrp2b-specific binding partner, Sema3fb, which we identified based on its expression in the OB. *Sema3fb* mRNA was detected in the dorsal OB with particularly high levels in the dorsal-medial area of the OB at 36hpf. This expression pattern fit with the potential function of Sema3fb as a repellent cue for Nrp2a or Nrp2b expressing OSNs. A likely null *sema3fb* mutant line (sa14466) was acquired, carrying a nonsense mutation that results in a premature stop codon. No increase in TRPC2- or OMP-class OSN axon misprojections were detected in *sema3fb* mutants (Fig. 4C). We also found no increase in the rate of TRPC2- or OMP-class OSN axon misprojections in *sema3fa*−/−;*sema3fb*−/− as compared to *sema3fa*−/− siblings (Fig. 4C). We conclude that *sema3fb* cannot account for the additional errors in *nrp2a*; *nrp2b* double as compared to *sema3fa* mutant embryos, and that additional unknown Nrp2 ligands remain to be identified.

### Both *nrp2a* and *nrp2b* act in the same pathway with *sema3fa*

**Fig. 5:**
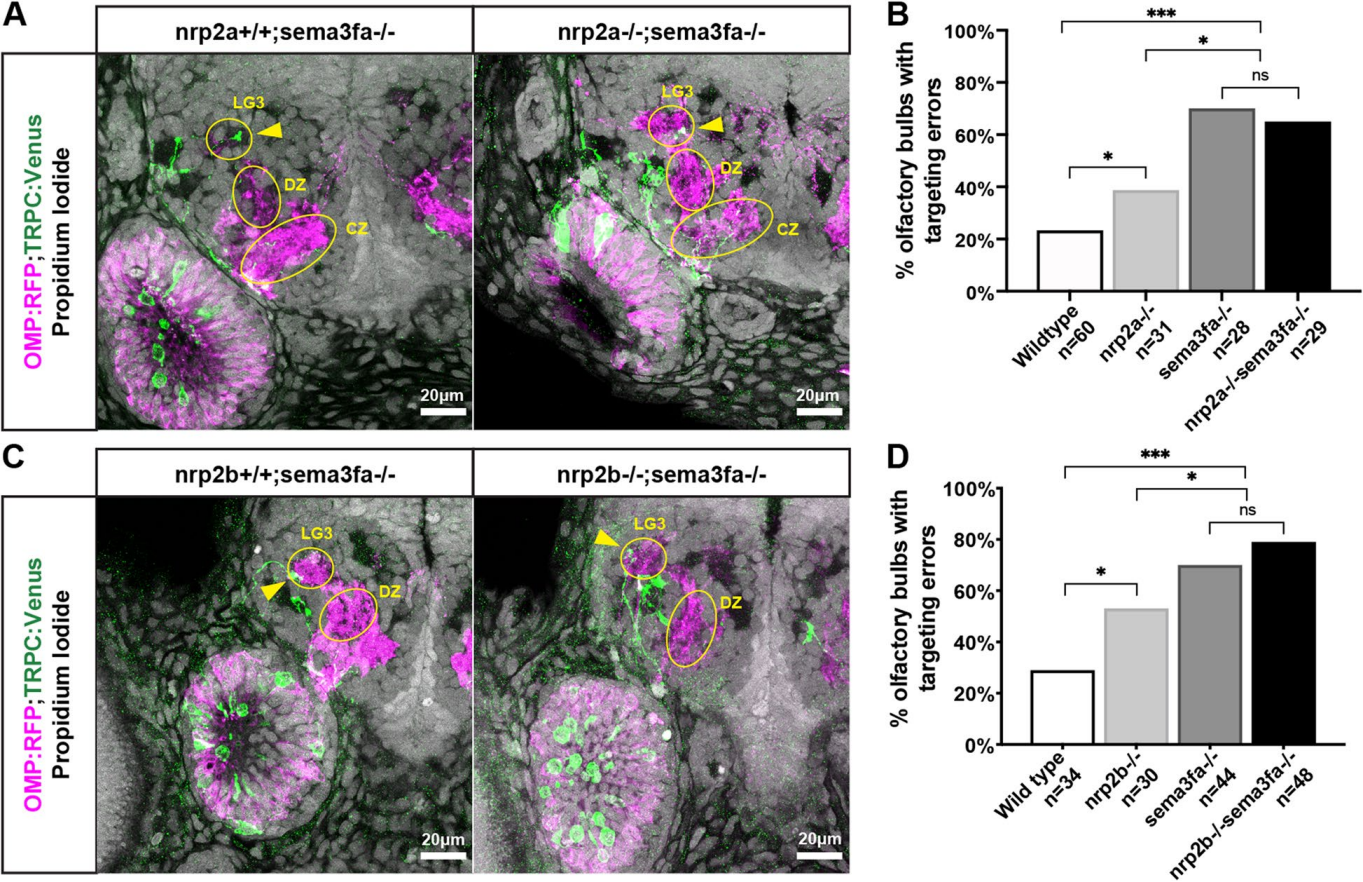
Both *nrp2a* and *nrp2b* act in the same pathway with *sema3fa.* **A.** Representative confocal sections of *sema3fa* mutant and *nrp2a;sema3fa* double mutant siblings. Yellow arrows indicate misprojecting axons. **B.** The misprojection phenotype in *nrp2a;sema3fa* double mutants is not significantly different from *sema3fa* mutant siblings. **C.** Representative confocal sections of *sema3fa* mutant and *nrp2b;sema3fa* double mutant siblings. Yellow arrows indicate misprojecting axons. **D.** The misprojection phenotype in *nrp2b;sema3fa* double mutants is not significantly different from *sema3fa* mutant siblings

The similar mutant phenotypes in *nrp2a*, *nrp2b*, and *sema3fa* mutants suggest that they may act in the same signaling pathway to regulate TRPC2-class OSN protoglomerular targeting. One prediction from this hypothesis is that if *nrp2a* and *sema3fa* act in the same pathway, the double mutant phenotype should be less severe than the effects of the two single mutant phenotypes added together. To test if this is true, we generated *nrp2a*+/−;*sema3fa*+/−; OMP:RFP;TRPC2:Venus fish. These animals were crossed to generate wild type, single mutant, and double mutant siblings for comparison. *Nrp2a* and *sema3fa* mutants from this cross had the expected TRPC2-class OSN misprojection phenotype observed previously, and *sema3fa* mutant animals had a slightly stronger misprojection phenotype as compared to *nrp2a* mutant animals. The phenotype of *nrp2a*−/−;*sema3fa*−/− fish was not significantly different from that of the *sema3fa*−/− mutant. Neither the number of misprojections nor the pattern of misprojections were significantly different between *sema3fa*−/− and *nrp2a*−/−;*sema3fa*−/− animals (Fig. 5A,B). Similarly, the number of misprojections and the pattern of misprojections were not significantly different between *sema3fa*−/− and *nrp2b*−/−;*sema3fa*−/− animals (Fig. 5C,D). These results are consistent with Sema3fa acting as a ligand for both Nrp2a and Nrp2b in guiding TRPC2-class OSN axons to their protoglomerular targets.

### Misprojecting growth cones fail to retract in *nrp2a* and in *sema3fa* mutants

One advantage of the zebrafish model system is the ability to examine the dynamics of developmental processes with live imaging. We took advantage of this to compare the development of OMP- and TRPC2-class projections in mutant larvae to heterozygote siblings. OMP:RFP; TRPC2:Venus embryos were mounted in agarose and imaged live between 30 hpf and 48 hpf. Spinning disk confocal microscopy generated a z-stack through each olfactory projection every 10 min during this 18-h period. This allowed us to visualize individual TRPC2-class axons and follow them as they projected into the olfactory bulb.

OMP:RFP and TRPC2:Venus expressing OSN axons have entered the OB at 30 hpf. OMP-class axons form a relatively compact bundle within the bulb. TRPC2-class axons occupy lateral and ventral-medial areas surrounding OMP axons. OMP- and TRPC2-class axons do not intermix freely. The relative organization of OMP and TRPC2 axons at this early stage is similar to that at 3 dpf, with the notable difference that protoglomeruli have not yet formed. As development continues, OMP-class axons separate into distinct clusters that become recognizable as the CZ, DZ, MG, and LG3 protoglomeruli at around 36 hpf. More TRPC2-class axons arrive in the OB and begin to form recognizable protoglomeruli by 38 hpf. Throughout this time period, TRPC2-class axons in particular can be seen dynamically extending and retracting in the OB, probing and retreating from areas larger than their ultimate protoglomerular targets.

**Fig. 6:**
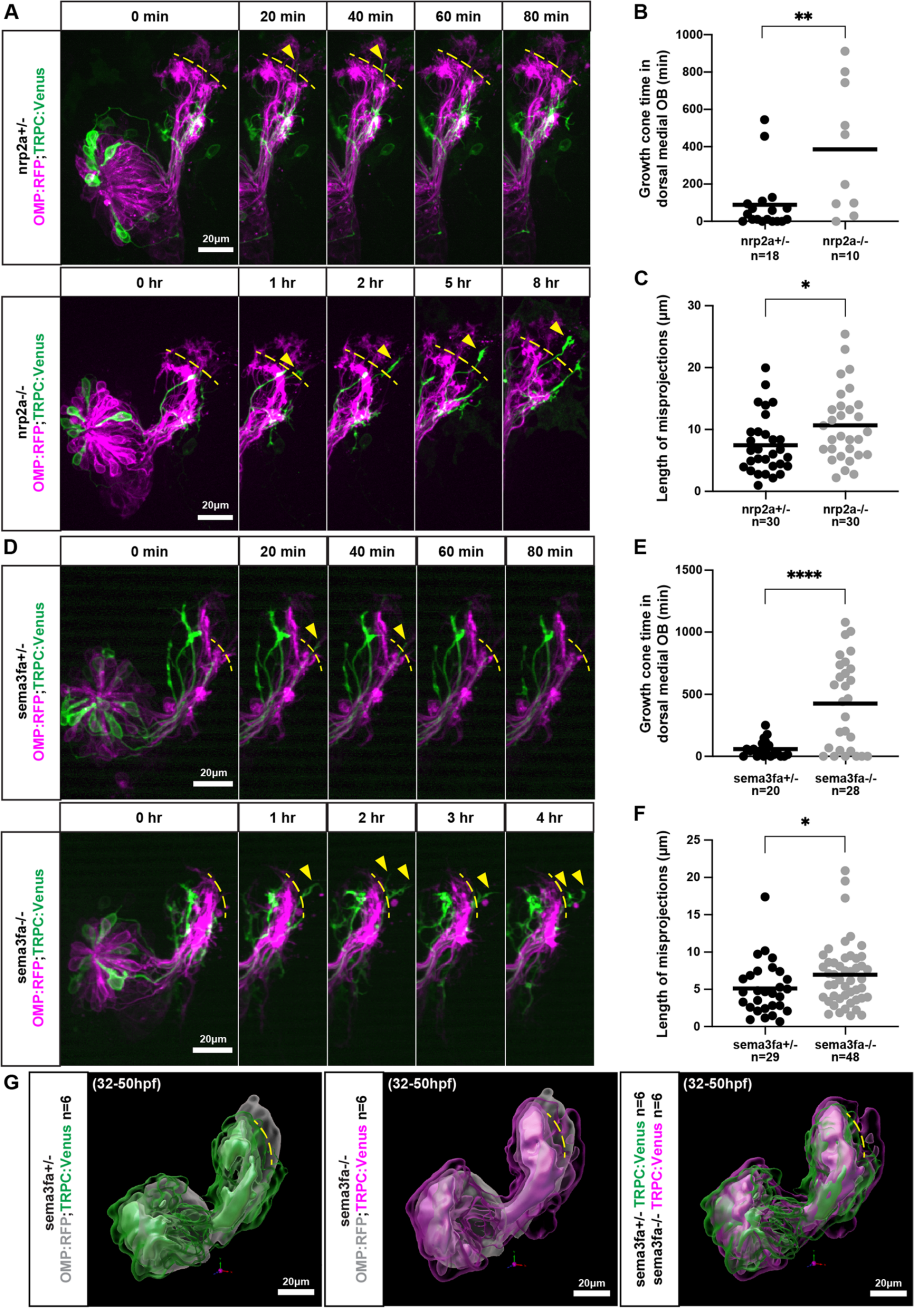
Misprojecting growth cones fail to retract in *nrp2a* and in *sema3fa* mutants. **A.** Live imaging sequences of *nrp2a* heterozygote and mutant siblings, showing misprojecting axons occupying the dorsal-medial OB. The yellow dotted lines indicate the dorsal boundary of the developing DZ and CZ protoglomeruli and denote the edge of the dorsal-medial OB region. Yellow arrows indicate misprojecting axons. **B.** The cumulative time that the dorsal-medial OB is occupied by TRPC2-class OSNs is greater in *nrp2a* mutants as compared to *nrp2a* heterozygous siblings. **C.** The maximum distance TRPC2-class axons project into the dorsal-medial OB is greater in *nrp2a* mutants as compared to heterozygotes. **D.** Live imaging sequences of *sema3fa* heterozygote and mutant siblings, showing misprojecting axons occupying the dorsal-medial OB. **E.** The cumulative time that the dorsal-medial OB is occupied by TRPC2-class OSNs is greater in *sema3fa* mutants as compared to heterozygous siblings. **F.** The maximum distance TRPC2-class axons project into the dorsal-medial OB is greater in *sema3fa* mutants as compared to heterozygotes. **G.** Model of average TRPC2-class axon locations in *sema3fa* heterozygotes and mutants during live imaging sequence. TRPC2-class axons are shown in green and magenta, and OMP-class axons in grey. The three TRPC2 surfaces encompass axon location probabilities, from most transparent to most opaque, of 5.6, 18.7, and 31.8%. Yellow dotted line represents the edge of the dorsal-medial OB region

To investigate whether the behavior of TRPC2-expressing OSN axons are affected in *nrp2a* mutants, *nrp2a*−/− animals were crossed to *nrp2a*+/− animals carrying OMP:RFP; TRPC2:Venus. The resulting embryos were live-imaged and subsequently genotyped after imaging. Just as in wild type fish, TRPC2-class axons are confined to ventral and ventral-lateral regions of the OB in *nrp2a*+/− embryos (Additional file 3). In *nrp2a*−/− embryos, we observed TRPC2-class axons entering into, and remaining within, the dorsal-medial region of the OB that is normally not occupied by TRPC2-class axons (Additional file 4). To quantify this phenotype, we defined the dorsal-medial OB as the region of the bulb dorsal to CZ and DZ protoglomeruli (Fig. 6, yellow dotted lines). In *nrp2a* mutant embryos, the time that TRPC2-class axons occupy the dorsal-medial OB is greater than in their *nrp2a*+/− siblings (386 vs 95.35 min; Fig. 6A,B). Axons also extend farther in *nrp2a*−/− as compared to heterozygous siblings, extending on average 10.65 μm past the CZ-D boundary compared to 7.47 μm (Fig. 6A,C).

The same experimental protocol was used to study *sema3fa* mutants. We observed an increased occupancy time in the dorsal-medial OB by TRPC2-class axons in *sema3fa* mutants as compared to heterozygous siblings (Additional files 5 and 6). The dorsal-medial OB was occupied on average 426.5 min in mutant animals as compared to 58.44 min in heterozygous siblings (Fig. 6D,E). The distance that misprojecting TRPC2-class axons extended past the CZ-DZ boundary was also greater in *sema3fa* mutants as compared to heterozygotes (Fig. 6D,F).

To get a better sense of the overall behavior of TRPC2-class axons in *sema3fa* heterozygotes vs mutants, we generated a 3D model of the live imaging dataset from six samples of each genotype (Fig. 6G). To create a probability map of axon locations, z-stacks of all 12 samples were aligned in 3D space. Each z-stack was converted to binary to normalize fluorescence intensity, and the resulting 3D time series were summed together to create an averaged time series. The time series was further summed along the time axis to create a 3D probability map of axon locations. Consistent with the manual quantification, comparing the 3D models for *sema3fa* heterozygotes to *sema3fa* mutants shows an increased probability of finding TRPC2 axons in the dorsal-medial OB (Fig. 6G).

We occasionally observed apoptosis of individual OSNs and the subsequent fragmentation of their corresponding axons (Additional files 4 and 5). Cell death was unlikely to have been induced by phototoxicity or other experimental procedures, since it was not increased by high laser illumination that caused widespread photobleaching of the fluorescent OSNs. Apoptosis was not observed more frequently in either *sema3fa* or *nrp2a* mutants as compared to heterozygote siblings, suggesting that cell targeting errors may not be corrected through cell death at this stage of olfactory circuit formation. Our results indicate that *nrp2a* and *sema3fa* are required early in the development of olfactory circuitry to exclude TRPC2-class axons from specific regions of the olfactory bulb and that these errors are not quickly corrected.

## Discussion

This study began with the observation that the RNAs for the axonal guidance receptors *nrp2a* and *nrp2b* are more highly expressed in TRPC2-expressing OSNs than in OMP-expressing OSNs, while *sema3fa* mRNA is well expressed in OMP expressing OSNs. As Sema3F is a known ligand for Nrp2 [51] and in most instances mediates repellent activity [50, 52], we hypothesized that the differential expression of these two signaling components may participate in the differential targeting of OMP- and TRPC2-class OSN axons to mutually exclusive protoglomeruli in the olfactory bulb. One mechanism through which this could occur is Sema3fs secreted by OMP-class processes repel TRPC2-class axons as they project towards and within the olfactory bulb. The two classes of axons are intermixed within the OE, but they separate as they enter the bulb. Fascicles of TRPC2-class axons surround a dense cable of OMP-class axons, like vines growing on the trunk of a tree. These separate TRPC2-class axon fascicles then terminate in ventromedial (OP) and dorsolateral (LG1, LG2, LG4, VPG) protoglomeruli that surround a cluster of OMP-class protoglomeruli (CZ, DZ, MG, LG3). If Sema3fa secreted from OMP-class axons helps to separate the two classes of axons, loss of *sema3fa* would be expected to induce ectopic fasciculation of TRPC2-class axons into OMP-class fascicles; no such mixing was detected. If another class 3 semaphorin functionally substitutes for *sema3fa*, mixing might be induced by the loss of *nrp2a*, *nrp2b*, or both; again, no such mixing was detected. These findings suggest that neither Sema3fa-nor Nrp2a/b-mediated signaling play a decisive role in the selective fasciculation of OMP- and TRPC2-class axons.

We find, however, that the exclusivity in protoglomerular targeting of OMP- and TRPC2-class axons to distinct and complementary protoglomeruli is degraded in the absence of Nrp2a, Nrp2b, or their repellant ligand Sema3fa. In these mutants TRPC2-class axons invade protoglomeruli that are normally exclusively occupied by OMP-class axons. Sema3fa produced by OMP-class OSNs and released from their processes may help to repel, and thereby exclude, Sema3fa-sensitive TRPC2-class axons from OMP-class protoglomeruli. This parallels findings in mice: VNO axons in the mouse, the class most similar to TRPC2-class OSNs in fish, occasionally peel off VNO axon fascicles and enter into and form glomeruli in ectopic Main Olfactory bulb territories in both *Sema3F* or *Nrp2* mutants [21, 22]. It has been hypothesized that in mouse, secreted semaphorins surrounding the vomeronasal nerve help promote its fasciculation in a Nrp2-dependent manner [22]. As we are proposing for the fish, Sema3F is thought to be secreted from Main Olfactory OSN axons in mice that are analogous to the OMP-class axons in fish [23]. Sema3fa/Nrp2 mediated repulsion cannot be the only mechanism that keeps OMP- and TRPC2-class axons from entering each other’s protoglomeruli in the fish, as there is limited mixing of the two classes of axons in either *sema3fa* mutants or in *nrp2a*; *nrp2b* double mutants.

A second striking axonal misguidance phenotype in *nrp2a*, *nrp2b*, or *sema3fa* mutants is an increase in TRPC2-class axons extending ectopically into the dorsal-medial region of the olfactory bulb. Both end-stage analysis and time-lapse studies show a higher probability of TRPC2-class axons occupying this region of the bulb in mutant embryos. One interpretation of this phenotype is that the expression of Nrp2 guidance receptors is required for TRPC2-class axons to detect repellent semaphorins expressed in the dorsal-medial OB. One of these repellents appears to be Sema3fa, since the same guidance phenotype is observed in *sema3fa* mutants. *Sema3fa* mRNA is detected along the anterior and dorsal-medial regions of the developing OB. In situ localization of other Nrp2 binding Sema3s (*sema3e*, *sema3fa*, *sema3fb*, *sema3ga*, *sema3h*) indicate that *sema3e* and *sema3fb* are also expressed in the dorsal-medial OB. We obtained a presumptive null *sema3fb* mutant line (Sanger sa14466) but did not observe increased ectopic TRPC2-class axon entry into the dorsal-medial OB in these mutant fish. We have not examined *sema3e* mutant fish.

*Nrp2* has been shown to be required for normal axon guidance of subsets of MOB type OSNs in both the mouse and fish [24, 25]. Nrp2-expressing OSNs may be restricted to the ventral-posterior region of the mouse MOB by responding to OSN-secreted Sema3F in the dorsal and anterior OB [23, 24]. OSN-secreted Sema3F also acts as a repellant cue for Nrp2-expressing mitral cells, olfactory projection neurons in the bulb [23]. In a previous study in the fish, loss of *nrp2b* induced misprojections of *or111–7* expressing OSN axons from their normal target, the Central Zone protoglomerulus, to the Dorsal Zone protoglomerulus [25]. As all OMP-class axons were uniformly labeled in the experiments in our study, we were not able to detect mistargeting of OSN subpopulations within specific OMP-class protoglomeruli. However, ectopic extension of OMP-class axons outside their normal protoglomeruli should be visible, and we did not detect any such events in *nrp2a*, *nrp2b*, or *nrp2a*;*nrp2b* double mutant embryos.

The zebrafish system is nearly ideal for following the early stages of olfactory circuit development in live preparations. In pioneering live imaging studies, Dynes and Ngai [11] visualized sparsely labeled fluorescent OSN axons at 1-h intervals. They concluded from their observations that OSN axons grow directly to their targets without stopping at intermediate targets and without sampling inappropriate target areas. With the more advanced microscopic techniques now available, we were able visualize a larger number of growing OSN axons in three dimensions at more frequent 10-min intervals, and as a consequence, were able to visualize the dynamic searching behavior of individual sensory axon growth cones. We observed the basal to apical migration of OSNs as they mature within the OE. We also noted the occasional apoptosis of OSNs along with the fracturing and degeneration of their axons as descried in previous studies [11]. Most importantly, we observed that individual OSN axons explore a larger area within the olfactory bulb outside their final target location. We observed individual TRPC2-class axons extending and retracting multiple times, sometimes extending the entire length of the OB and retracting back to more proximal locations within an hour. TRPC2-class axons can therefore sample large areas before settling on a defined target. In either *nrp2a* or *sema3fa* mutants, TRPC2-class axons extended within and occupied the dorsal-medial OB for greater periods of time than in heterozygous animals. In the heterozygotes, the axons that extend into this region quickly retract proximally, while in mutant animals, TRPC2-class axons explore this space for longer periods. Lack of either *nrp2a* or *sema3fa* appears to prolong the duration of TRPC2-class occupation of the dorsal-medial OB, likely through the removal of a repellent signaling pathway that would normally chase them from the region. This live imaging approach increases the sensitivity of our mutant phenotype analysis. It also suggests that altered axon dynamics could be a useful marker for future investigation of OSN axon guidance factors.

A small number of TRPC2-class OSNs have misprojecting axons in either *nrp2a* or *nrp2b* mutants. These axons erroneously project into OMP-specific protoglomeruli or misproject dorsally into non-protoglomerular regions of the OB. This misprojection phenotype is much more severe and is fully penetrant in *nrp2a*;*nrp2b* double mutant animals. Our results imply that *nrp2a* and *nrp2b* receptors play partially redundant but largely parallel roles in the same guidance process. We cannot be certain that the sensitivity of *nrp2* mRNAs by in situ detection provides a truly reliable picture of physiologically relevant levels of *nrp2* expression. It is therefore possible that both *nrp2s* are expressed in most or all TRPC2 class neurons. It is formally possible that the two *nrp2s* work best together in a heterodimeric complex and less efficiently in homodimeric complexes, accounting for the partial redundancy of function between the two. More likely, however, is that each *nrp2* contributes to the pathfinding abilities of a distinct but overlapping population of OSNs. The non-overlapping subset of each population may be selectively affected by the loss of one or the other *nrp2*, while all cells in the two populations would be affected by the loss of both *nrp2a* and *nrp2b*. This interpretation is consistent with our detection of *nrp2a* and *nrp2b* mRNAs in overlapping subsets of OSNs.

The early teleost genome is posited to have undergone a duplication event subsequent to its divergence from terrestrial vertebrates [27]. As a consequence, zebrafish possess two paralogs of *nrp2* and several of the class 3 semaphorins. Many paralogous genes are expressed in divergent expression patterns in the embryonic fish. As a rule, each semaphorin paralog has a conserved neuropilin binding profile as compared to either its paralog or its corresponding mouse ortholog [28]. It is unclear to what degree each paralog is specialized in its developmental function. In this study, we find that the two *nrp2* paralogs have similar guidance functions for a distinct subset (TRPC2-class) of sensory neurons. Interestingly, this is not the case for the two *sema3f* paralogs. This suggests that in practice paralogs can either work semi-redundantly, as the do the nrp2s, or a more specialized and independent fashion, as do the sema3fs.

Partial functional redundancy, as for example between *nrp2a* and *nrp2b*, between axonal guidance receptors expressed in overlapping populations of neurons would be expected to provide some level of robustness in neuronal circuit formation. A nearly identical principal has been elegantly expounded for the determination of neuronal identity [16]. In both cases, no single gene would be decisive, as outcomes would depend on the effects of several genes with similar functions. In our experience, loss of any single guidance cue or receptor has thus far produced relatively low frequencies of targeting errors in the developing zebrafish olfactory system, while as expected, error frequencies increase when two guidance-related genes are knocked out together. As this study and others [25, 26] have shown, loss of *nrp1a*, *nrp1b*, *nrp2a*, *nrp2b*, *robo2*, *robo3*, *sema3d*, or *dcc* all induce reliable but relatively low levels of guidance errors. The simultaneous loss of *nrp2b* and *sema3d*, *nrp1a* and *dcc*, or *nrp2a* and *nrp2b* induce more frequent errors in the trajectories of the same axons than either single mutant alone. It is easy to picture the sequential contributions that multiple axonal guidance cues make as an axon extends toward its target, encountering different cues as it advances [53, 54]. In addition, many cues may simultaneously compete or cooperate to fine-tune pathway or target selection. The multiplicity of cues acting on a growth cone would provide some degree of robustness and also a mechanism for evolutionary change. Projections could be fine-tuned or made more complex through the addition of guidance signals to those already in place. The differential expression of duplicated semaphorin and neuropilin genes in the zebrafish provide a fertile substrate for an evolutionary fine-tuning process.

## Conclusion

We conclude that *nrp2a*, *nrp2b*, and *sema3fa* are each required for the exclusion of TRPC2-class OSNs from OMP-specific protoglomeruli in the developing olfactory bulb. This function is mediated through a shared pathway with *sema3fa* likely acting as a repellent ligand for TRPC2-class OSNs expressing *nrp2a* and/or *nrp2b*, altering growth cone behavior and constraining the sample space of TRPC2-class OSN axons. *Nrp2a* and *nrp2b* play partially redundant and parallel roles in this context. The relative severity of misprojection phenotypes suggests that additional cues, likely other class-3 semaphorins, act through *nrp2a* and *nrp2b* to affect TRPC2-class axon pathfinding. These findings are consistent with a model in which a robust, partially redundant mechanism of multiple cues cooperate to influence the trajectory of individual axons, without a single cue being decisive.

## Supplementary Information

**Additional file 1.** 3D projection of a z-stack of a wild type 72 hpf olfactory bulb. Green is TRPC2: Venus and Magenta is OMP:RFP. Z-stack was taken at 1 μm intervals.

**Additional file 2. **3D model of *sema3fa* and *sema3fb* mRNA distribution within the OB at 36 hpf. Magenta spheres represent *sema3fa* mRNA, cyan spheres represent *sema3fb* mRNA, green structure represents OMP:Gal4;UAS:Citrine expressing OSNs, and grey structure represents the OB.

**Additional file 3. **Maximum projection of time-lapse live imaging z-stack showing a *nrp2a*+/− olfactory hemisphere. Green is TRPC2: Venus and Magenta is OMP:RFP. Time-lapse starts at 32 hpf and continues for 680 min, at 10 min intervals. Z-stack was taken at 1 μm intervals.

**Additional file 4. **Maximum projection of time-lapse live imaging z-stack showing a *nrp2a*−/− olfactory hemisphere. Green is TRPC2: Venus and Magenta is OMP:RFP. Time-lapse starts at 32 hpf and continues for 680 min, at 10 min intervals. Z-stack was taken at 1 μm intervals.

**Additional file 5. **Maximum projection of time-lapse live imaging z-stack showing a *sema3fa*+/− olfactory hemisphere. Green is TRPC2: Venus and Magenta is OMP:RFP. Time-lapse starts at 32 hpf and continues for 680 min, at 10 min intervals. Z-stack was taken at 1 μm intervals.

**Additional file 6. **Maximum projection of time-lapse live imaging z-stack showing a *sema3fa*−/− olfactory hemisphere. Green is TRPC2: Venus and Magenta is OMP:RFP. Time-lapse starts at 32 hpf and continues for 680 min, at 10 min intervals. Z-stack was taken at 1 μm intervals.

**Additional file 7: Supplemental figure 1. **Quantification of misprojections.** A.** Pattern of misprojections of TRPC2: Venus expressing OSNs in *nrp2a* mutants and wild type siblings. **B.** Pattern of misprojections of TRPC2: Venus expressing OSNs in *nrp2b* mutants and wild type siblings. **C.** Pattern of misprojections of TRPC2: Venus expressing OSNs in *sema3fa* mutants and wild type siblings. **D.** Pattern of misprojections of TRPC2: Venus expressing OSNs in *nrp2a*;*nrp2b* double mutants, *nrp2a* single mutants, *nrp2b* single mutants, and wild type siblings. **E.** Pattern of misprojections of TRPC2: Venus expressing OSNs in *nrp2a*;*sema3fa* double mutants, *nrp2a* single mutants, *sema3fa* single mutants, and wild type siblings. **F.** Pattern of misprojections of TRPC2: Venus expressing OSNs in *nrp2b*; *sema3fa* double mutants, *nrp2b* single mutants, *sema3fa* single mutants, and wild type siblings.

## Data Availability

The datasets used and/or analyzed during the current study are available from the corresponding author on reasonable request.

## Abbreviations

CZ: Central zone
dpf: Days post fertilization
DZ: Dorsal zone
hpf: Hours post fertilization
LG1: Lateral glomerulus 1
LG2: Lateral glomerulus 2
LG3: Lateral glomerulus 3
LG4: Lateral glomerulus 4
MG: Medial glomerulus
OB: Olfactory bulb
OE: Olfactory epithelium
OP: Olfactory plexus
OR: Odorant receptor
OSN: Olfactory sensory neuron
VPG: Ventral posterior glomerulus
