# Correction to: Loss of Neuropilin2a/b or Sema3fa alters olfactory sensory axon dynamics and protoglomerular targeting

**DOI:** 10.1186/s13064-022-00160-w

**Published:** 2022-03-05

**Authors:** Ryan P. Cheng, Puneet Dang, Alemji A. Taku, Yoon Ji Moon, Vi Pham, Xiaohe Sun, Ethan Zhao, Jonathan A. Raper

**Affiliations:** Department of Neuroscience, University of Pennsylvania School of Medicine, Philadelphia, PA 19104 USA

**Correction to: Neural Dev 17, 1 (2022)**

10.1186/s13064-021-00157-x

Following publication of the original article [1], the last two figures in the paper were misordered during the publication process and references to one of the figures were omitted. The figure entitled “Both nrp2a and nrp2b act in the same pathway with sema3fa” formerly appeared as Fig. 6 and now appears correctly as Fig. 5. The figure entitled “Misprojecting growth cones fail to retract in nrp2a and in sema3fa mutants” formerly appeared as Fig. 5 and now appears as Fig. 6. The correct figures are given below. Text referring to the corrected Fig. 5 was amended to: “Neither the number of misprojections nor the pattern of misprojections were significantly different between sema3fa−/− and nrp2a−/−;sema3fa−/− animals (Fig. 5A,B). Similarly, the number of misprojections and the pattern of misprojections were not significantly different between sema3fa−/− and nrp2b−/−;sema3fa−/− animals (Fig. 5C,D). Also, the following typographical errors, TRCP2 and TPRC2 on page 14 and TRCP2 on figure 6 caption were amended to TRPC2. This version of the paper now reflects these corrections to the original.

**Fig. 5:**
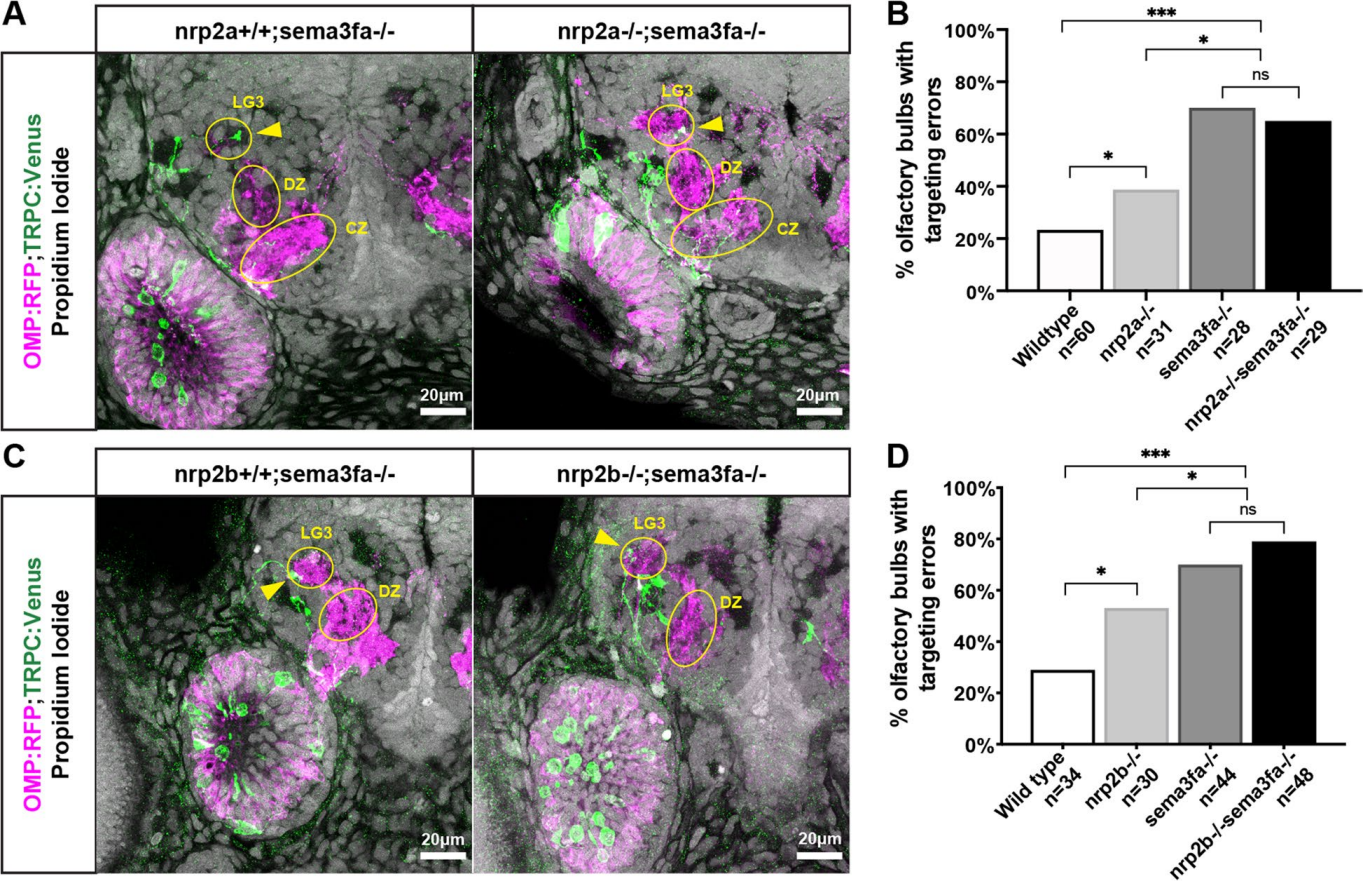
Both *nrp2a* and *nrp2b* act in the same pathway with *sema3fa****.*** **A** Representative confocal sections of *sema3fa* mutant and *nrp2a;sema3fa* double mutant siblings. Yellow arrows indicate misprojecting axons**. B** The misprojection phenotype in *nrp2a;sema3fa* double mutants is not significantly different from *sema3fa* mutant siblings. **C** Representative confocal sections of *sema3fa* mutant and *nrp2b;sema3fa* double mutant siblings. Yellow arrows indicate misprojecting axons**. D** The misprojection phenotype in *nrp2b;sema3fa* double mutants is not significantly different from *sema3fa* mutant siblings. Model of average TRPC2-class axon locations in *sema3fa* heterozygotes and mutants during live imaging sequence. TRPC2-class axons are shown in green and magenta, and OMP-class axons in grey. The three TRCP2 surfaces encompass axon location probabilities, from most transparent to most opaque, of 5.6, 18.7, and 31.8%. Yellow dotted line represents the edge of the dorsal-medial OB region

**Fig. 6:**
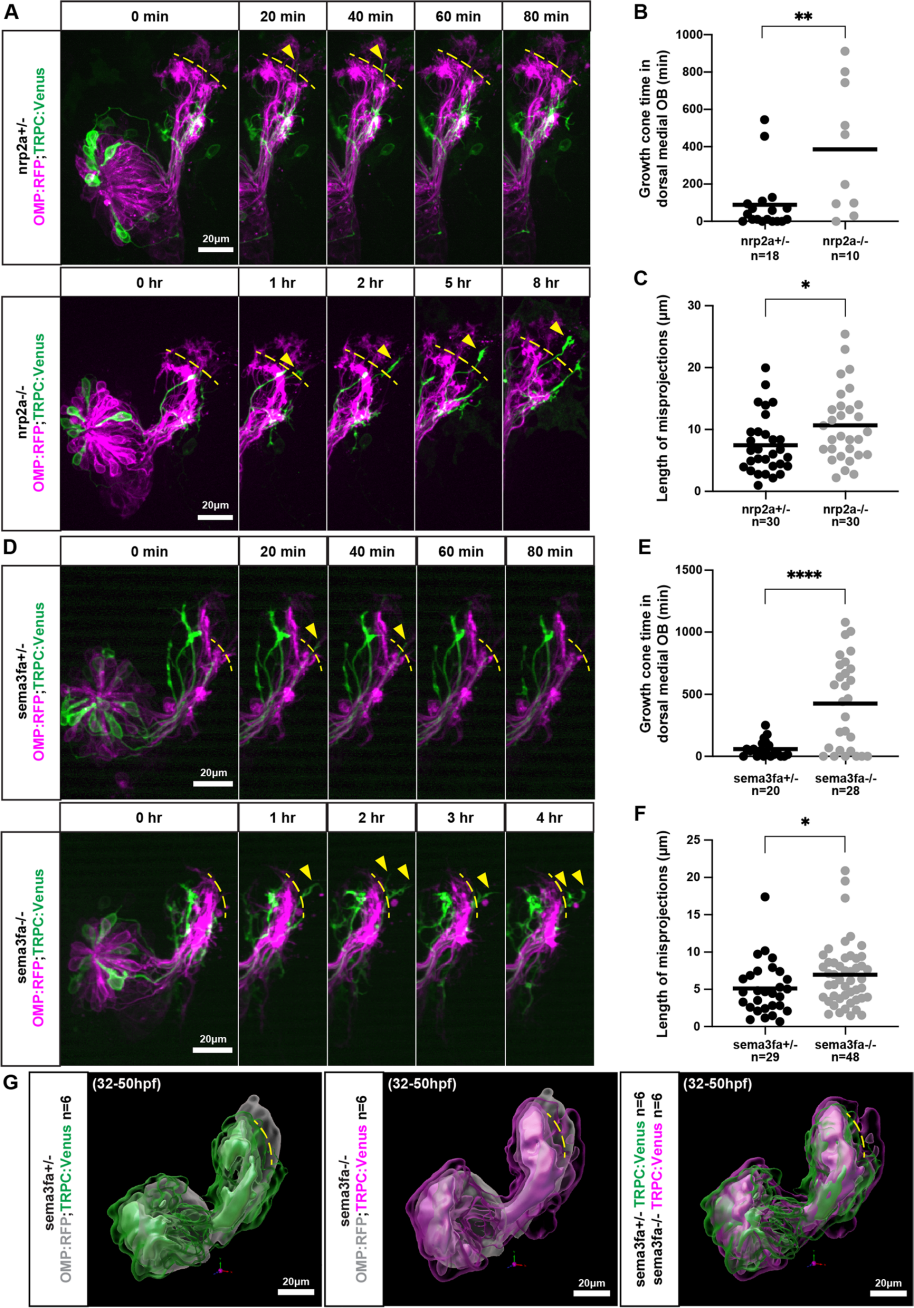
Misprojecting growth cones fail to retract in *nrp2a* and in *sema3fa* mutants. **A** Live imaging sequences of *nrp2a* heterozygote and mutant siblings, showing misprojecting axons occupying the dorsal-medial OB. The yellow dotted lines indicate the dorsal boundary of the developing DZ and CZ protoglomeruli and denote the edge of the dorsal-medial OB region. Yellow arrows indicate misprojecting axons. **B** The cumulative time that the dorsal-medial OB is occupied by TRPC2-class OSNs is greater in *nrp2a* mutants as compared to *nrp2a* heterozygous siblings. **C** The maximum distance TRPC2-class axons project into the dorsal-medial OB is greater in *nrp2a* mutants as compared to heterozygotes. **D** Live imaging sequences of *sema3fa* heterozygote and mutant siblings, showing misprojecting axons occupying the dorsal-medial OB. **E** The cumulative time that the dorsal-medial OB is occupied by TRPC2-class OSNs is greater in *sema3fa* mutants as compared to heterozygous siblings. **F** The maximum distance TRPC2-class axons project into the dorsal-medial OB is greater in *sema3fa* mutants as compared to heterozygotes. **G** Model of average TRPC2-class axon locations in sema3fa heterozygotes and mutants during live imaging sequence. TRPC2-class axons are shown in green and magenta, and OMP-class axons in grey. The three TRPC2 surfaces encompass axon location probabilities, from most transparent to most opaque, of 5.6, 18.7, and 31.8%. Yellow dotted line represents the edge of the dorsal-medial OB region

The original article [1] has been corrected.

## References

[1] Cheng RP, Dang P, Taku AA (2022). Loss of Neuropilin2a/b or Sema3fa alters olfactory sensory axon dynamics and protoglomerular targeting. Neural Dev.

