# Supplementary figures and images for: Loss of Neuropilin2a/b or Sema3fa alters olfactory sensory axon dynamics and protoglomerular targeting
Source: Neural Dev. 2022 Jan 3;17:1. doi: 10.1186/s13064-021-00157-x (PMC8725463; doi:10.1186/s13064-021-00157-x)

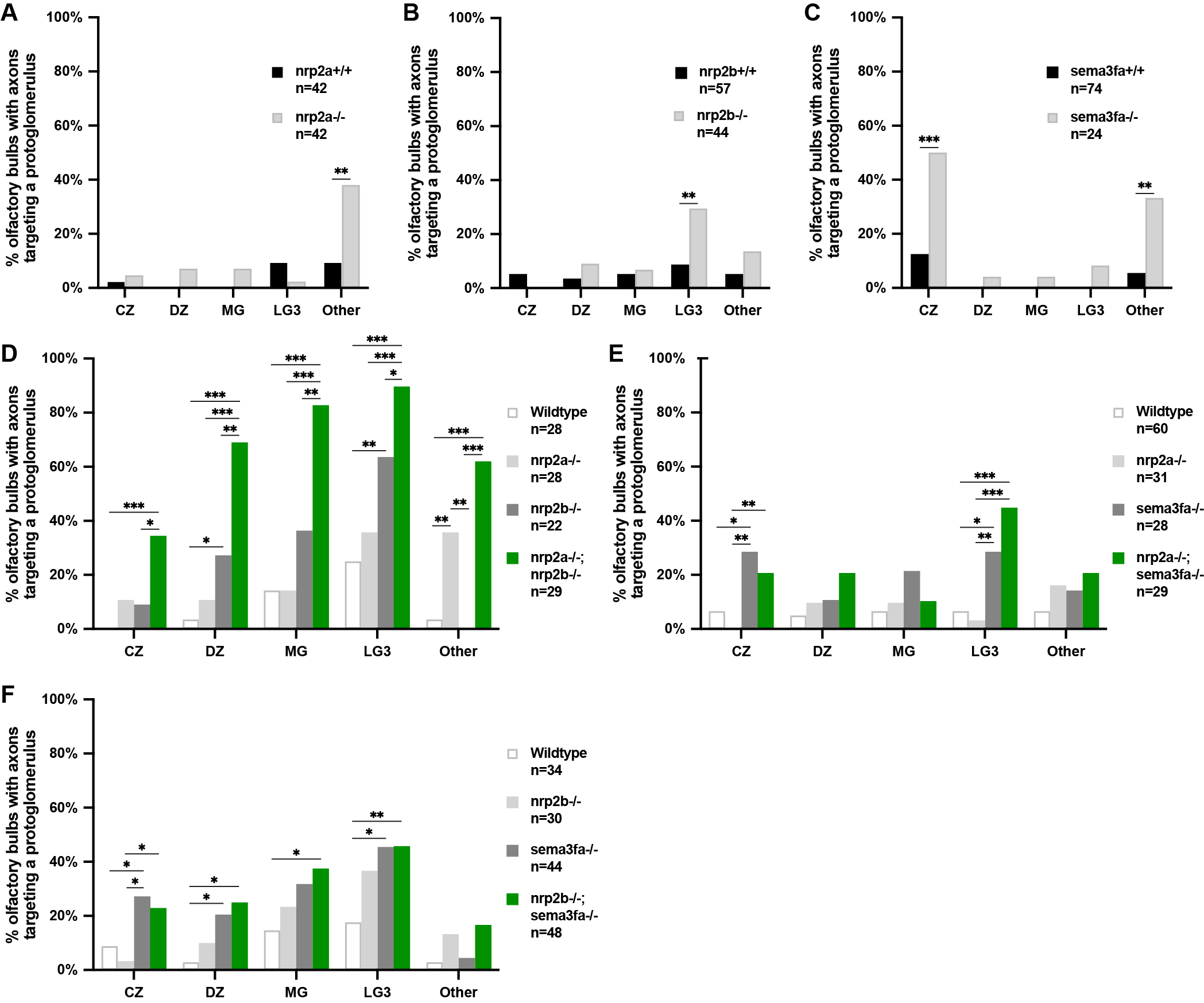

Supplement: Supplementary file 7 — Additional file 7: Supplemental figure 1. Quantification of misprojections. A. Pattern of misprojections of TRPC2: Venus expressing OSNs in nrp2a mutants and wild type siblings. B. Pattern of misprojections of TRPC2: Venus expressing OSNs in nrp2b mutants and wild type siblings. C. Pattern of misprojections of TRPC2: Venus expressing OSNs in sema3fa mutants and wild type siblings. D. Pattern of misprojections of TRPC2: Venus expressing OSNs in nrp2a;nrp2b double mutants, nrp2a single mutants, nrp2b single mutants, and wild type siblings. E. Pattern of misprojections of TRPC2: Venus expressing OSNs in nrp2a;sema3fa double mutants, nrp2a single mutants, sema3fa single mutants, and wild type siblings. F. Pattern of misprojections of TRPC2: Venus expressing OSNs in nrp2b; sema3fa double mutants, nrp2b single mutants, sema3fa single mutants, and wild type siblings. [file 13064_2021_157_MOESM7_ESM.tif]
